# Phage Selective Pressure Reduces Virulence of Hypervirulent *Klebsiella pneumoniae* Through Mutation of the *wzc* Gene

**DOI:** 10.3389/fmicb.2021.739319

**Published:** 2021-10-06

**Authors:** Lingjie Song, Xianggui Yang, Jinwei Huang, Xiaokui Zhu, Guohui Han, Yan Wan, Ying Xu, Guangxin Luan, Xu Jia

**Affiliations:** ^1^Non-coding RNA and Drug Discovery Key Laboratory of Sichuan Province, Chengdu Medical College, Chengdu, China; ^2^Department of Laboratory Medicine, Clinical Medical College and the First Affiliated Hospital of Chengdu Medical College, Chengdu, China; ^3^Department of Respiratory Diseases, Sixth Affiliated Hospital of Wenzhou Medical University, Lishui, China

**Keywords:** bacteriophage, hypervirulent *K. pneumoniae*, *Phage resistance*, virulence, *wzc*

## Abstract

Hypervirulent *Klebsiella pneumoniae* (hvKp), one of the major community-acquired pathogens, can cause invasive infections such as liver abscess. In recent years, bacteriophages have been used in the treatment of *K. pneumoniae*, but the characteristics of the phage-resistant bacteria produced in the process of phage therapy need to be evaluated. In this study, two Podoviridae phages, hvKpP1 and hvKpP2, were isolated and characterized. *In vitro* and *in vivo* experiments demonstrated that the virulence of the resistant bacteria was significantly reduced compared with that of the wild type. Comparative genomic analysis of monoclonal sequencing showed that nucleotide deletion mutations of *wzc* and *wcaJ* genes led to phage resistance, and the electron microscopy and mucoviscosity results showed that mutations led to the loss of the capsule. Meanwhile, animal assay indicated that loss of capsule reduced the virulence of hvKp. These findings contribute to a better understanding of bacteriophage therapy, which not only can kill bacteria directly but also can reduce the virulence of bacteria by phage screening.

## Introduction

*Klebsiella pneumoniae*, a Gram-negative bacterium, is one of the most important opportunistic nosocomial pathogens. Generally, according to their virulence, *K. pneumoniae* has been broadly classified into two main groups, classic *K. pneumoniae* (cKp), and hypervirulent *K. pneumoniae* (hvKp). HvKp has the ability to cause life-threatening, community-acquired infections, including liver abscesses complicated by endophthalmitis, meningitis, osteomyelitis, and necrotizing fasciitis, in young and healthy individuals and is therefore associated with high morbidity and mortality (Shon et al., 2013). The K1, K2, K20, K54, and K57 capsular serotypes *K. pneumoniae* are considered to be the main hypervirulent strains (Russo et al., 2018; Wei et al., 2021). In recent years, multidrug-resistant hypervirulent strains have mainly emerged around the Asian Pacific Rim (Gu et al., 2018; Russo and Marr, 2019), creating a new challenge in combating this already dangerous pathogen. Therefore, it is highly desirable to choose an alternative treatment to replace or complement classic approaches with antibiotics.

Bacteriophage therapy is one of the most promising potential options (Nobrega et al., 2015; Bao et al., 2020). For conventional antibacterial agents, one of the main research focuses with phage therapy is phage-resistant bacterial variants (Oechslin, 2018). It is essential to understand more the mechanisms underlying the resistance to phages in order to monitor or prevent this phenomenon. The mechanisms of phage resistance, including preventing phage adsorption, preventing phage DNA entry, and cutting phage nucleic acids, are complex and varied (Labrie et al., 2010). The blocking of phage adsorption receptors is the most important evasive strategy for *K. pneumoniae* (Domenico et al., 1994; Paczosa and Mecsas, 2016), of which capsules act as phage adsorption receptors in this bacterium, similar to *Escherichia coli* and *Acinetobacter baumannii* (Clarke et al., 2000; Popova et al., 2017).

Interestingly, capsular polysaccharide, a polysaccharide matrix that coats the cell, has been identified as a virulence factor (Paczosa and Mecsas, 2016). In bacteria, the capsule confers resistance against the bactericidal activity of antimicrobial peptides, complement, and phagocytes (Domenico et al., 1994; Paczosa and Mecsas, 2016) and is synthesized by gene products from the capsular polysaccharide synthesis (*cps*) locus. Among these capsular-related genes, *wzc* encodes protein and exhibits autophosphorylation protein tyrosine kinase (TK) activity. In addition, the initial glycosyltransferase (GTs) encoded by *wbaP* and *wcaJ* are involved in the synthesis of the capsular repeat and further catalyzed by specific (non-initial) GTs allowing the addition of sugars (Tan Y. H. et al., 2020). In *K. pneumoniae cps* gene cluster, the deletion or mutation of *wzi, wza, wzb*, and *wzc* genes has a significant impact on virulence (Ernst et al., 2020; Niu et al., 2020). Based on these studies, here we hypothesized that the loss of the capsule could cause the strain to develop phage resistance, with a consequent decrease in virulence. To verify this conjecture and explore the mechanism, we conducted further studies.

In the present study, two bacteriophages against K57 capsular serotype *K. pneumoniae* were isolated from the sewage, and *in vitro* and *in vivo* treatment experiments were performed to evaluate the virulence of the mutant strains resistant to these two bacteriophages using the *Galleria mellonella* model. Sequence analysis revealed that two genes, *wzc* and *wcaJ*, had mutations, and the roles of *wzc* in phage-resistant mutant strains were fully investigated.

## Materials and Methods

### Bacteria Strains and Growth Conditions

*K. pneumoniae* strains isolated from four hospitals in China were used in this study (Table 1). All strains were identified by 16S rDNA polymerase chain reaction (PCR) (listed in Supplementary Table 1) and stored at –80°C in 15% (vol/vol) glycerol, and all culturing was carried out in lysogeny broth (LB) at 37°C with shaking at 200 revolutions/min.

**TABLE 1:** Host range of phages.

**Strain**	**K type**	**MLST**	**hvKpP1**	**hvKpP2**	**Source**
hvKpLS7 (host)	K57	ST412	√	√	a
WCHKP030925	K1	ST23	—	—	b
hvKpLS8	K2	ST65	—	—	a
355	K64	ST11	—	—	c
356	K64	ST11	—	—	c
366	K150	ST2325	—	—	c
377	K84	ST485	—	—	c
070869	K57	ST412	√	√	d
072117	K57	ST218	√	√	d
0717609	K57	ST218	√	√	d
072179	K57	ST412	√	√	d
XJ-K1	K64	ST11	—	—	c
XJ-K2	K64	ST11	—	—	c

*a, Sixth Affiliated Hospital of Wenzhou Medical University; b, West China Hospital; c, General Hospital of Western Theater Command of PLA; d, Clinical Medical College and the First Affiliated Hospital of Chengdu Medical College.*

### Phage Isolation

Phages were isolated from a local wastewater station in Chengdu, Sichuan. Briefly, untreated sewage was mixed with hvKpLS7 culture at a volume ratio of 1:1. The enrichment culture was then incubated overnight at 37°C, centrifuged (5,000 × *g*, 5 min), and the supernatant filtered through a 0.22-μm filter to remove cells. This filtrate was mixed with hvKpLS7 in molten semisolid soft agar (0.7% agar) and poured over solidified 1.5% nutrient agar plates. All overlay agar plates were allowed to set and then incubated overnight at 37°C. The resulting plaques were subjected to three rounds of plaque purification. The propagation of bacteriophages was determined according to the protocols of the Erna Li laboratory (Li et al., 2016). Purified phages were stored at 4°C in SM buffer (100 mM NaCl, 8 mM MgSO_4_ ⋅ 7H_2_O, and 50 mM Tris-HCl at pH 7.5), and the titer was determined by the double-layer agar assay.

### Transmission Electron Microscopy of Phage

Phage particles were spotted onto a carbon-coated copper grid and negatively stained with 2% (wt/vol) phosphotungstic acid. After drying, phages were observed on a Tecnai G^2^ F20 electron microscope (FEI, United States) operated at 80 kV to acquire morphological information of single-phage particles.

### Thermal and Acid–Base Stability Tests

Thermal and acid–base stability tests were performed as previously described (Jurczak-Kurek et al., 2016), with some modifications. The phage solution was diluted with SM buffer, after which the phage was treated at a specified temperature or pH for 1 h. After treatment, the titer of the phage was determined by double-layer agar plate method. The results were expressed as phage stability in terms of the percentage of initial viral counts.

### Adsorption Experiments and One-Step Growth Analysis

Phage adsorption to host bacteria was performed as described previously (Kropinski et al., 2009), with minor modifications. The host strains were cultured to the logarithmic growth phase, mixed with phage liquid with a multiplicity of infection (MOI) of 0.1. The mixture was incubated at 37°C for and centrifuged (13,000 × *g*, 1 min) immediately. The titer of the supernatant was determined using the method described above, and the phage adsorbed to the host bacteria was calculated based on the titer.

For the one-step growth analysis, the host bacteria were cultured to the logarithmic stage, and phage liquid was added at an MOI of 10, incubated at 37°C for 10 min, and centrifuged (13,000 × *g*, 1 min, 4°C). The supernatant was discarded, and the sediment was resuspended in liquid LB medium. The bacterial suspension was adjusted to 10^8^ colony-forming units (CFUs)/mL. The liquid was incubated with rotary shaking (200 rpm, 37°C), and aliquots of 100 μL were sampled from time 0 to 1 h with 10-min intervals. The titer was determined using the double-layer agar plate method.

### Host Range Determination

The bacteria listed in Table 1 were used for host range analysis by standard spot tests (Kutter, 2009). Briefly, strains were grown overnight in an LB medium. Suspension (10 μL) purified phage suspension containing 10^7^ plaque-forming units (PFUs)/mL were spotted in the middle of a lawn of bacteria and left to dry before overnight incubation. Lysis characteristics were established at the spot where the phage was deposited. The assay host refers to the tested *K. pneumoniae* clinical isolates, and the isolation host refers to the *K. pneumoniae* isolate hvKpLS7, with which we initially isolated the phage.

### Sequencing and Analysis of Bacteriophage Genomes

Bacteriophage DNA was prepared from high-titer phage preparations with phenol–chloroform and sodium dodecyl sulfate (SDS) (Lu et al., 2013). Briefly, the lysis buffer [20 mM EDTA, 50 μg/mL proteinase K, and 0.5% (wt/vol) SDS in SM solution] was added to the purified phages stock solution. The mixture was incubated at 56°C for 1 h, after which an equal volume of phenol–chloroform–isoamyl alcohol (25:24:1) was added, followed by centrifugation at 12,000 × *g* for 10 min. The aqueous layer was extracted with chloroform at 10,000 × *g* for 10 min. The aqueous layer was collected, mixed with 400 μL of isopropanol, and then stored at –20°C for 1 h. The mixture was centrifuged at 4°C and 12,000 × *g* for 10 min, and the precipitated DNA was collected by sterile double distilled water.

DNA samples were sequenced in the second generation by Ion S5 (Thermo Fisher Scientific, United States) and third-generation by MinION (Oxford Nanopore Technologies, United Kingdom) genome sequencer. The sequencing data were assembled using the SPAdes assembler v. 3.13.2 (Bankevich et al., 2012), HGAP4, and Canu v1.6. The MUMmer v3 (Delcher et al., 2003) software was used to analyze the contigs obtained by splicing the second- and third-generation sequencing data to reconfirm the assembly results and determine the positional relationship between the contigs and to determine the gap between contigs.

The complete genomes of phages were sequenced and analyzed using a variety of bioinformatics tools. Open reading frames (ORFs) were predicted using SoftBerry.^1^ Genome annotations were checked through sequence comparison of protein sequences using the blastn software.^2^ Genome comparative analysis was performed using Easyfig.

### Bacteriophage Therapy Assay and Determination of Virulence of Strains

The *G. mellonella* model was used to evaluate the antibacterial efficacy of bacteriophages *in vivo* and the virulence of *K. pneumoniae* strains (Gu et al., 2018; Lu et al., 2018). All the injections were carried out into the last left proleg by use a Hamilton syringe (Insua et al., 2013). The minimum lethal concentration of *K. pneumoniae* infection by larval caterpillars was determined to be 10^7^ CFUs/mL within 3 days. When larvae did not respond to touch, they were considered dead. In the *in vivo* experiment, the larvae were divided into four groups, with 20 randomly chosen larvae used for each group: (i) only injected with 10 μL phosphate-buffered saline (PBS), (ii) injected with 10 μL of 10^6^ CFUs/mL host bacteria, (iii) injected only with 10 μL of 10^7^ PFUs/mL of phage, and (iv) injected with 10 μL of 10^6^ CFUs/mL host bacteria and then injected with 10 μL of 10^7^ PFUs/mL phage within 30 min. All larvae were incubated at 37°C, and the number of dead larvae was counted at 12-h intervals up to 72 h after the incubation.

Virulence determination tests were performed according to the bacterial concentration in the previous phage treatment experiment (10^7^ CFUs/mL). Sixteen *G. mellonella* larvae were injected with 10 μL of the inoculum in every group. Survival was analyzed by Kaplan–Meier analysis with a log–rank test; differences were considered statistically significant at *p* < 0.05.

### Screen for Phage-Resistance Strains

Phages hvKpP1 and hvKpP2 were mixed with the host bacteria for cultivation, and the mixture was cultured using double-layer soft agar. Plates were incubated overnight at 37°C, and the resulting colonies were picked up and saved for further assays.

### Bacteria Growth Curves

All strains were cultured as described above. The following day, cultures were incubated in LB at a concentration of 1 × 10^7^ CFUs/mL and added to individual wells of a 96-well microtiter plate. Plates were incubated for 12 h at 37°C, and absorbance readings at 600 nm were recorded every 30 min using BMG SPECTROstar^®^ Nano. Growth rates of the bacterial strains were calculated using three biological replicates.

### Phage Adsorption Efficiency Assay

Phage adsorption efficiency assays were performed as previously described, with modest modifications (Cai et al., 2018). Briefly, 2 mL late-exponential-phase culture of each *K. pneumoniae* strain (1 × 10^9^ CFUs/mL) was mixed with 10 μL diluted phage (approximately 1 × 10^6^ PFUs), and the mixture was incubated for 10 min at 37°C with shaking. The mixture was centrifuged, and then the supernatant was filtered through 0.22-μm filters, and phage particles in filtrates were quantitatively analyzed by plaque assay in triplicate. The phage adsorption efficiency was calculated using the equation [(initial titer – residual titer in the supernatant)/initial titer] × 100%.

### Mucoviscosity Assay

The mucoviscosity of the capsule was assessed by low-speed centrifugation of the liquid culture (Bachman et al., 2015). Various overnight cultures of *K. pneumoniae* were grown to adjust to OD_600_ of 1 and centrifuged at 1,000 × *g* for 5 min. The OD_600_ values of the supernatants were then measured.

### Macrophage Phagocytosis Assay

RAW264.7 cells were seeded in 24-well plates and grown in Dulbecco modified eagle medium (10% fetal bovine serum, 100 mg/mL ampicillin, and 100 mg/mL streptomycin) at 37°C and 5% CO_2_. *K. pneumoniae* strains were added at an MOI of 10 bacteria per host cell, and the inoculum was plated for CFUs. Cells were rinsed three times with PBS and then incubated on fresh medium containing 300 μg/mL gentamicin to kill extracellular bacteria. After three washes, cells were lysed with 0.1% TritonX-100 for 20 min, diluted, and plated for bacterial CFU enumeration. The percentage of phagocytosed bacteria per inoculum was calculated and normalized to that of hvKpLS7. Three biological replicates per strain were used for each experiment.

### Transmission Electron Microscopy of Bacteria

Transmission electron microscopy (TEM) was performed using JEM-1400PLUS (JEOL, Japan). Bacterial samples were prepared as described previously and modified (Ernst et al., 2020). Briefly, samples were fixed for at least 2 h at room temperature in 3% glutaraldehyde and postfixed with 1% osmium tetroxide, dehydrated in alcohol grades, incubated with propylene oxide, and infiltrated overnight in a 1:1 mixture of propylene oxide and epoxy low-viscosity resin. The following day, samples were embedded in epoxy resin and polymerized. Ultrathin sections (approximately 50 nm) were cut on a Reichert EM UC7 microtome, transferred to copper grids stained with lead citrate, and examined using a JEM-1400PLUS TEM, and images were recorded.

### Bacterial Genome Sequencing and Analysis

Genomic DNA of wild-type (WT) hvKpLS7 and phage-resistant mutants were sequenced at Sangon Biotech (Shanghai, China) using the Illumina HiSeq platform (∼1 Gbp/sample, paired-end) as previously described (Tan D. et al., 2020). The quality of raw sequencing reads was evaluated using FastQC (Brown et al., 2017). Low-quality reads and adapter sequences were trimmed using Trimmomatic software (Bolger et al., 2014). Following the Genome Analyzer Toolkit (GATK) best practices pipeline (McKenna et al., 2010), the genomic mapping tool Burrows–Wheeler Aligner was used to map low-divergent sequences (Li and Durbin, 2010) to the reference genome of *K. pneumoniae*. Mutations, including base substitutions, deletions, and insertions, were detected using SAMtools, MarkDuplicates, and BEDTools (Li et al., 2009; Quinlan and Hall, 2010). DNA deletion mutations were further validated by PCR and sequencing.

### Cloning and Complementation

Genomic DNA from hvKpLS7 was used as the template for WT gene cloning *via* PCR; the primes are listed in Supplementary Table 1. PCR products were purified and cloned into the pBAD24-CM vector by homologous recombination using the ClonExpress II One Step Cloning Kit (Vazyme, Nanjing, China). Recombinant plasmids were first heat-shocked into *E. coli* DH5α and further electroporated into corresponding phage-resistant mutants. The complementation strains were verified by PCR and sequenced using pBAD24 primers. The bacterial isolates transformed with an empty vector were tested in parallel.

### Statistical Analysis

All experiments were performed with *n* equal to 3. Statistical analysis was performed using GraphPad Prism v.6.0 (Software Inc., La Jolla, CA, United States) to plot. For all phage adsorption efficiency, mucoviscosity, and macrophage phagocytosis assays, comparisons between mutant and WT, and WT and complementation strains were evaluated for statistical significance using the one-way analysis of variance (ANOVA). The survival curves with the Kaplan–Meier method followed a log-rank test to calculate the differences in survival. Statistical significance was set at *p* < 0.05.

## Results

### Phage Isolation and Host Range

**FIGURE 1:**
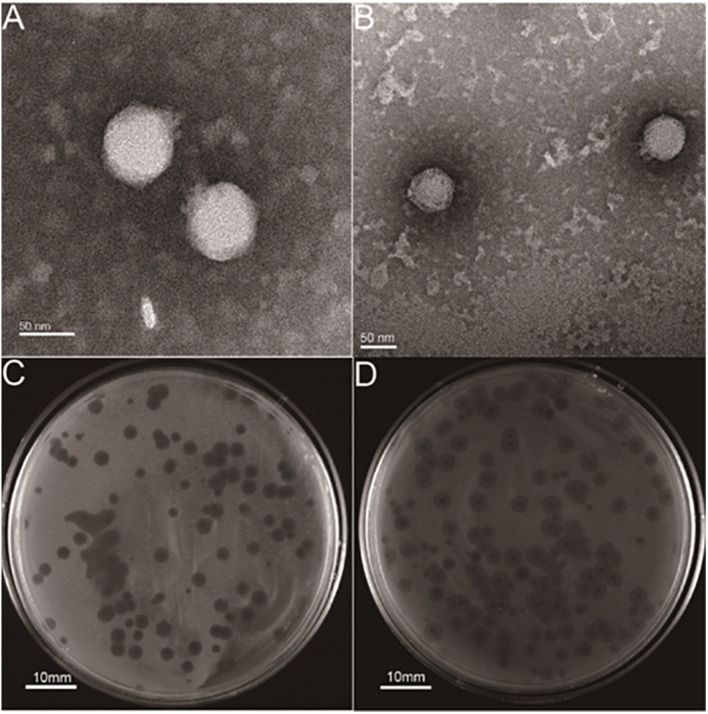
Structural characterization and plaque morphology of isolated phages. TEM of phage **(A)** hvKpP1 and **(B)** hvKpP2. The bar indicates 50 nm. Plaques of phage **(C)** hvKpP1 and **(D)** hvKpP2 on *K. pneumoniae* hvKpLS7. The bar indicates 10 mm.

Two lytic phages, vB_KpnP_cmc20191 (referred to as hvKpP1) and vB_KpnP_cmc20192 (hvKpP2), were isolated from sewage; microscopic observation of virion morphology by TEM showed that the phages were classified as members of the Podoviridae family (Figures 1A,B). They formed different plaques on the bacterial lawn of *K. pneumoniae* strain hvKpLS7 (Figures 1C,D). hvKpP1 produced clear plaques, whereas hvKpP2 formed smaller lytic center plaques surrounded by a semitransparent halo. The clinical host strain hvKpLS7 was characterized as an hvKP by 11 virulence-associated genes, including a*erobactin*, *iroN*, *rmpA*, *rmpA2*, *ybtS*, *ureA, wabG*, *ycf*, *entB*, *iutA*, and *fimH* (Zhan et al., 2017), and was further confirmed in the *G. mellonella* model, using hypervirulent WCHKP030925 (Feng et al., 2018) as a positive control (Supplementary Figure 1). This clinical strain belonged to the K57 capsular serotype and host range experiments also confirmed that only strains belonging to the K57 capsular serotype were specifically targeted by both phages; others, including K1, K2, K64, K150, and K84, were not; this further confirmed that infections by phages had a relationship with the K capsular serotype (Table 1).

### Phage Characterization

To determine phage stability, the sensitivity of phages to temperature and pH stability was analyzed (Table 2). Phages were stable in the pH range of 4–11 or under 70°C (Supplementary Figures 2A,B). These results are in line with those of previous studies (Ciacci et al., 2018; Teng et al., 2019), showing that these two phages can maintain high lytic activity under broad physicochemical conditions. The adsorption rate curves of the two phages showed that more than 90% of bacteriophages were adsorbed within 10 min (Supplementary Figure 2C). According to the one-step growth curves (Supplementary Figure 2D), the replication cycle of hvKpP2 was approximately 60 min. However, the latent period of hvKpP1 was relatively short, approximately only 30 min. The burst size of hvKpP1 (149 PFUs/cell) was greater than that of hvKpP2 (96 PFUs/cell). The eclipse period and burst size may be part of the reason for the difference in plaque production between the two phages. A summary comparison of these two phages is presented in Table 2.

**TABLE 2:** Summary phage comparison.

**Parameter**	**hvKpP1**	**hvKpP2**
Genome length (bp)	44,066	44,314
GC content (%)	53.9	54
**Plaque morphology**		
Lytic center	+++	+
Halo	–	+++
**Growth kinetics**		
Latent period, min	10	10
Burst size, PFUs, means (SD)	149 (6)	98 (10)
**Stability range**		
Temperature, °C	≤60	≤60
ph	4–11	4–11

*+ and +++ denote the relative size of the attribute; – denotes an attribute that is not visibly appreciable.*

**FIGURE 2:**
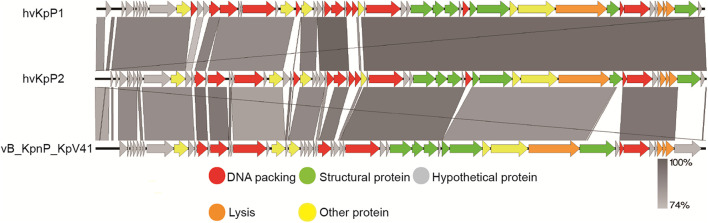
Pairwise BLASTn comparison of hvKpP1, hvKpP2, and vB_KpnP_KpV41. The genome map was performed using the Easyfig. Arrows represent predicted ORFs; the direction of the arrow represents the direction of transcription. Different colors denote different functional groups of bacteriophage genes.

Bioinformatics analysis helps us to better understand and predict biological characteristics of phages. Thus, the complete genomes of the two phages were sequenced and analyzed using bioinformatics tools. The genome of phage hvKpP1 was 44,066 bp in length with 54% G + C content, whereas hvKpP2 was 44,314 bp in length with 53.9% G + C content. In total, 55 and 58 putative coding regions (CDSs) were detected by RAST analysis. Importantly, the lack of lysogeny, host conversion, and toxins supported the growth kinetics data, suggesting that the two phages possess lytic properties and could be used for therapeutic purposes (Philipson et al., 2018). Nucleotide BLAST analysis revealed that hvKpP1 and hvKpP2 exhibited high DNA similarity. We compared their genomes using Easyfig. As shown in Figure 2, the main difference between hvKpP1 and hvKpP2 lies in the genes encoding the DNA packaging. Among them, hvKpP1 contains more genes encoding HNH family proteins, which are considered to be related to phage DNA replication in reference research (Kala et al., 2014). This explains that hvKpP1 achieved a greater burst size compared with hvKpP2. The two phages had high homology in the tail packaging region, indicating that their adsorption targets for the host bacteria were the same. The complete nucleotide sequences of phages vB_KpnP_cmc20191 and vB_KpnP_cmc20192 were determined and deposited in GenBank under accession numbers MT559526 and MT559527, respectively.

### *In vivo* Efficiency of Bacteriophage Treatment

**FIGURE 3:**
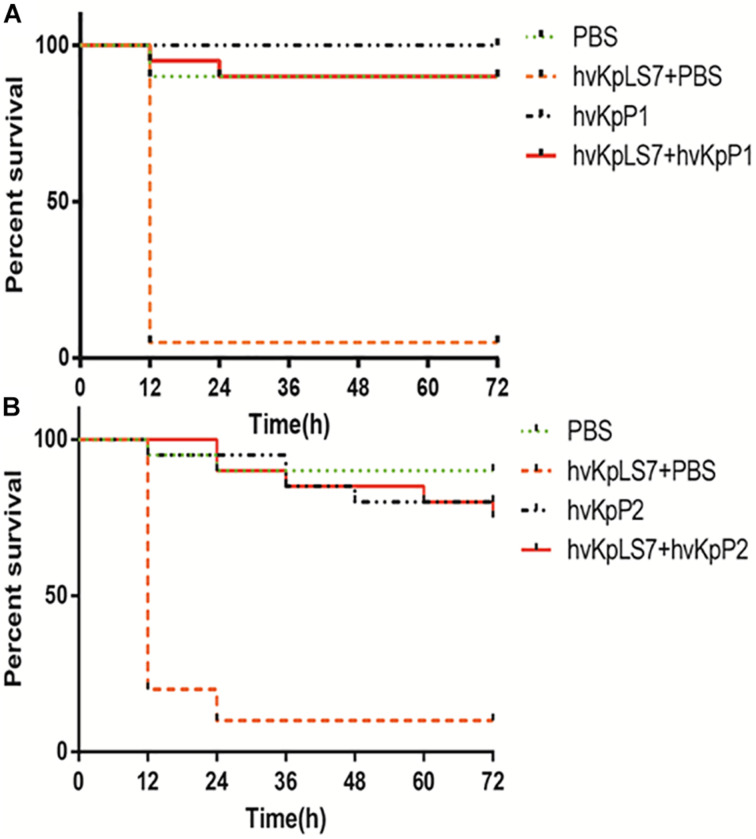
Survival of *G. mellonella* larvae after infection by hvKp hvKpLS7 and phage therapy. Treatment by **(A)** hvKpP1 and **(B)** hvKpP2.

The efficacy of phages hvKpP1 and hvKpP2 was evaluated *in vivo* using the *G. mellonella* larvae model. For larva infected by the host strain hvKpLS7, the survival rate was only 5 and 10% in 3 days, respectively. In the phage treatment groups, survival was significantly superior, with 3-day survival rates of 75 and 90%. There was a significant difference in the survival rates between the larva infection group and the treatment group (*p* < 0.05). Additionally, the larvae group injected only with phages still had a high survival rate, demonstrating the safety of the phages in this model (Figure 3). The present study reports, for the first time, on phage efficacy against K57 capsular serotype *K. pneumoniae* in *G. mellonella.*

### Characterization of Phage-Resistance *K. pneumoniae*

**FIGURE 4:**
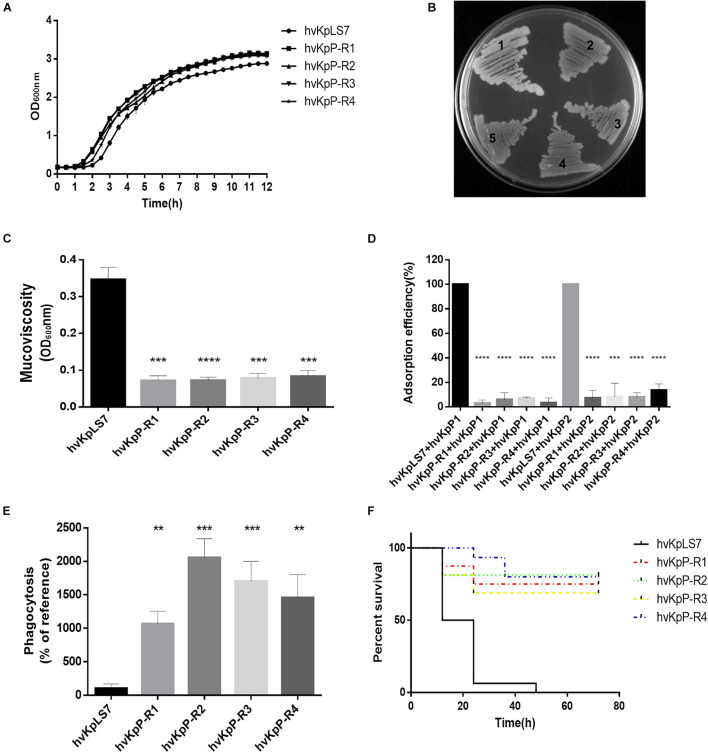
Characterization of phage-resistant *K. pneumoniae*. **(A)** Growth curves of the WT and mutant of *K. pneumoniae* strains. **(B)** Morphology comparison between colonies of *K. pneumoniae* on an LB agar plate. hvKpLS7 (mucoid, moist, and sticky) and phage-resistant mutant (dry, rough, and transparent). **(C)** Centrifugation analysis of hvKpLS7, hvKpP-R1, hvKpP-R2, hvKpP-R3, and hvKpP-R4. **(D)** Adsorption efficiencies of hvKpP1 and hvKpP2 binding to *K. pneumoniae* hvKpLS7, hvKpP-R1, hvKpP-R2, hvKpP-R3, and hvKpP-R4. **(E)** Phagocytosis by RAW264.7 macrophages. **(F)** Survival of *G. mellonella* larvae. The one-way ANOVA test was performed to determine statistically significant differences between each mutant and hvKpLS7. **p < 0.01, ***p < 0.001, ****p < 0.0001.

**FIGURE 5:**
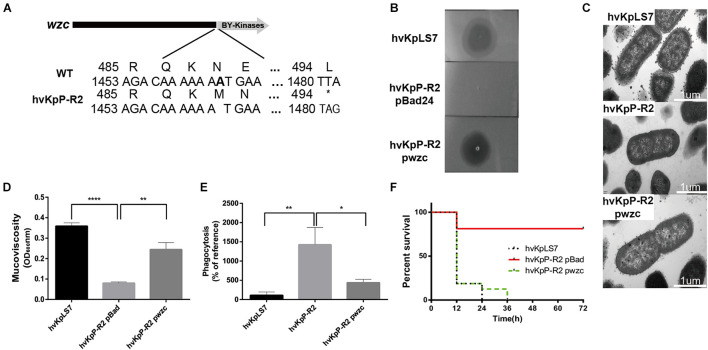
Sensitivity and virulence of the *wzc* mutant restore phage by complementation. **(A)** Schematic representation of *wzc* in *K. pneumoniae* hvKpLS7 and hvKpP-R2. Genes are represented as arrows. **(B)** Spot test assay of phage on the parental *K. pneumoniae* strain hvKpLS7 and its derived mutants (hvKpP-R2 pBad and hvKpP-R2 pwzc). **(C)** The *wzc* gene affects capsule production. TEM of WT, phage-resistant, and complementation strain. For every isolate, one representative image from six images obtained from one section is shown. **(D)** Mucoviscosity is restored in hvKpP-R2 pwzc. **(E)** Phagocytosis by RAW264.7 macrophages of WT, phage-resistant, and *wzc* gene complementation strain. **(F)** Survival rates of *G. mellonella* with hvKpLS7, hvKpP-R2 pBad, and hvKpP-R2 pwzc. One-way ANOVA was performed to determine statistically significant differences. *p < 0.05, **p < 0.01, ****p < 0.0001.

Although phage treatment in the *G. mellonella* model showed excellent results, phage-resistant *K. pneumoniae* colonies were easily produced on bacterium–phage cocultured LB agar plates. On cocultured LB plates with hvKp (1 × 10^6^ CFUs) and phages (1 × 10^7^ PFUs), the frequencies of these phage-resistant mutants were 5 × 10^–5^ ± 1 × 10^–5^. We are interested in whether these phage-resistant *K. pneumoniae* would have an impact on phage therapy. To test the difference between WT and phage-resistant *K. pneumoniae*, four mutants were randomly selected from the plates named hvKpP-R1, hvKpP-R2, hvKpP-R3, and hvKpP-R4. Growth curves of phage-resistant strains and the WT indicated that resistance to phage had no significant effect on their growth (Figure 4A). On the LB agar plates, the WT host hvKpLS7 was moist, hypermucoid, and reflective when photographed, whereas the phage-resistant bacteria had a translucent appearance and a reduced ability to produce mucoid (Figure 4B). All the mutant strains showed negative string test results (results are not shown). Such changes have also been reported by Tan D. et al. (2020). Some studies have suggested that hypermucoviscous is related to the capsule (Walker et al., 2019); hence, mucoviscosity of the capsule was assessed by low-speed centrifugation of the liquid culture. Strains were grown in LB, diluted to an optical density at 600 nm (OD_600_) of 1, and then subjected to low-speed centrifugation. During the centrifugation process, hvKpLS7 did not sediment well, the supernatant was still turbid, and the OD_600_ of the supernatant was 0.35. In contrast, the phage-resistant bacteria were well precipitated in the low-speed centrifugation experiment, the supernatant was relatively clear, and the average OD_600_ decreased to 1/3, compared with the WT (Figure 4C). This indicated that *K. pneumoniae* with phage resistance significantly decreased capsular adhesion. TEM images in subsequent experiments also showed that isolates were capsule deficient (Figure 5C). The bacterial capsule was reported as the primary receptor for phages (Bertozzi Silva et al., 2016), so the adsorption efficiency of these mutant strains was conducted. The adsorption efficiency of hvKpP1 and hvKpP2 to selected phage-resistant mutants was significantly lower than that of WT (Figure 4D). Based on the above experimental data, the selected strains could not be adsorbed by phages because of the lack of ability to produce capsule, which made the strains resistant to phages.

Previous studies have demonstrated that *K. pneumoniae* capsules confer significant phagocytosis resistance to macrophages (Fang et al., 2004). These strains were coincubated with phagocytes RAW264.7, the lysates of washed phagocytes were daubed on agar plates, and the bacterial colonies were counted and recorded. Results showed that the amount of the mutant strain devoured by the phagocytes increased at least 10 times compared with that of the WT, indicating that the phagocytes could effectively eliminate these mutant bacteria (Figure 4E).

Furthermore, the *G. mellonella* infection model was used to assess whether phage-resistant bacteria resulted in changes in the virulence. As the same concentration of bacteria, the survival rate with 3 days of these mutants (12/16, 13/16, 11/16, and 12/16) was significantly higher than that of hvKpLS7 (0/16) for the infection (Figure 4F). These results demonstrated that the phage-resistant *K. pneumoniae* reduced their virulence, possibly owing to the absence of a capsule.

### Identification of Mutant Genes in Phage-Resistant Strains

To identify the genes responsible for resistance in bacteria, genomes of WT hvKpLS7 and phage-resistant mutants were sequenced using the Illumina HiSeq platform and comparatively analyzed. High-probability mutations (defined as high-frequency, non-silent mutations within an ORF) were selected for further validation.

Through comparative analysis of genome sequencing, among the four resistant strains screened, there were no differences in genomic detected between hvKpP-R1 and WT hvKpLS7; however, the strain showed similar characteristics to the rest of the mutants. We speculate that this may be epigenetically driven (Cota et al., 2015) and warrants further investigation. While hvKpP-R2 and hvKpP-R4 have mutations in the *wzc* gene, the mutation of hvKpP-R2 is the frameshift mutation of *wzc* gene caused by the deletion of base A in position 1,463 (Figure 5A), whereas hvKpP-R4 is the premature translation termination of the coding gene caused by the A→C transversion at position 2,163. In addition, hvKpP-R3 sequencing analysis revealed that four bases between position 908 and 911 were missing, and so the translation of the *wcaJ* was terminated prematurely. To further assess the ratio of *wzc* and *wcaJ* mutations in phage-resistant isolates, 41 resistant clones were randomly selected for PCR and sequencing analysis of *wzc* and *wcaJ* genes. The results showed that 17 clones were *wzc* mutations, and 13 clones were *wcaJ* mutations (Supplementary Table 1). Of the 17 strains in which the *wzc* gene was mutated, nine contain the T deletion at nucleotide 1,118; two contain the T substitution for the C at position 253, all resulting in premature termination. In addition, there are four strains with a deletion A in position 1,273 and 1,463, the 27–30 fragment, or the 807–895 fragment, respectively, leading to frameshift of the gene. Among the 13 strains with *wcaJ* mutations, 10 isolates were due to a frameshift by the deletion of AT at position 910–911, two strains were the mutation of T to C substitution at position 1,268 that resulted in the substitution of amino acid proline to leucine, and one strain was the premature termination due to the substitution of T by a at position 903. Impressively, the simultaneous occurrence of both *wzc* and *wcaJ* mutations has not been observed within the same strain. Additionally, all of the isolates could be complemented. Both *wzc* and *wcaJ* are located on clusters associated with capsule synthesis. The role of the *wcaJ* gene in phage resistance and virulence has been reported (Cai et al., 2019; Tan D. et al., 2020); therefore, here we focus on the *wzc* gene.

### The *wzc* Mutant Restores Phage Sensitivity and Virulence by Complementation

The *wzc* gene belongs to the capsular polysaccharide gene clusters, which facilitates polymerization of capsular polysaccharide when activated by its TK domain (Wugeditsch et al., 2001). As shown in Figure 5A, the deletion of base A resulted in a frameshift of the *wzc* gene in hvKpP-R2, which causes the coding gene to terminate prematurely at amino acid 494; therefore, the mutation resulted in a missing TK domain and loss of catalytic activity. To confirm that the identified mutation was necessary for the phage recognition and bacteria virulence, we transform a plasmid containing WT *wzc* of the *K. pneumoniae* strain hvKpLS7 into hvKpP-R2, designated hvKpP-R2 pwzc.

Spot test results indicated that the strain hvKpP-R2 pwzc restored phage sensitivity with a smaller semitransparent halo (Figure 5B). TEM analysis showed that the boundaries of hvKpP-R2 were smoother than those of hvKpLS7 and hvKpP-R2 pwzc, which confirmed that phage-resistant selection caused the loss of the capsule, and the expression of recombinant *wzc* regained the polymerization of the capsule significantly (Figure 5C). Mucoviscosity assay showed that hvKpP-R2 pwzc recovered the mucoviscosity of the capsule (Figure 5D). Moreover, the phagocytic capacity of macrophages against the mutant complementation was decreased (Figure 5E). According to the 3-day survival rate of *G. mellonella*, results of the hvKpP-R2 pwzc and WT infection groups were similar (Figure 5F), indicating that after complementing the *wzc* gene of the WT strain, the virulence of this mutant strain was restored.

## Discussion

HvKp usually infects healthy individuals in the community. In recent years, the emergence of carbapenem-resistant hvKp has been regarded as a serious threat to public health. In this era of emerging antibiotic resistance, phage therapy, as a personalized treatment, provides a new option for patients who did not respond to antibiotics alone. Although phages have been used clinically (Corbellino et al., 2020; Qin et al., 2020), a detailed understanding of phage biology is required. In this study, two Podoviridae phages, hvKpP1 and hvKpP2, were isolated and characterized. The phages identified in the present study differed from other *K. pneumonia*e phages found in previous reports (Tan et al., 2019; Teng et al., 2019; Zhang et al., 2020). The phylogenetic tree shows that these phages are distinct from each other (Supplementary Figure 3). However, their hosts were all hypervirulent strains, suggesting that the capsule may be a universal phage receptor for hvKp. The phages in this study can lyse the K57 capsular-type hypervirulent *K. pneumonia* specifically. K57 *K. pneumoniae* (K57-KP) was also considered a highly virulent *K. pneumoniae* in clinical investigations (Qu et al., 2015; Solovieva et al., 2018). Bioinformatics analysis showed that these phages do not contain lysogeny, host conversion, or toxin genes, suggesting that the two newly discovered phages were eligible for the phage therapy candidates.

During phage therapy, phage-resistant bacteria have been previously reported (Filippov et al., 2011; Gordillo Altamirano et al., 2021). Indeed, phage-resistant mutations are easily manipulated *in vitro*. Generally, phage-resistant bacteria are thought to be negative to phage therapy. However, recent studies have demonstrated that phage-resistant bacteria many times also have a positive side. Resistant strains reduced the level of bacterial resistance in *K. pneumoniae* by expelling multidrug resistance clusters or plasmids (Majkowska-Skrobek et al., 2021). *A. baumannii* resensitized to human complement, β-lactam antibiotics through loss of function of capsular genes in the pressure selection of phages (Gordillo Altamirano et al., 2021). Here we found that the mutant survived at the pressure of bacteriophages at the cost of virulence reduction in hvKp, consistent with previous reports (Cai et al., 2019). The hvKp is notable for high virulence, and the capsule was considered as the main virulent factor in hvKp. WT hvKp could escape the phagocytosis of immune cells by removing the capsule, much like a Gecko breaks its tail to survive. The naked mutant has no coat to remove, and so it is easily cleaned up by macrophages. On the other hand, our research shows that the capsule is the main receptor of these phages, and its absence will significantly reduce the adsorption efficiency. The serotypes of *K. pneumoniae* are mainly classified according to different capsular types, and the phages in this study specifically lyse K57-type *K. pneumoniae*, further confirming that the capsule plays a key role in phage recognition.

Capsular polysaccharides are synthesized by the *wza-wzb-wzc* system. The *wzc* phosphorylates an endogenous UDP-glucose dehydrogenase (Ugd) involved in the production of exopolysaccharide colanic acid, as well as the production of UDP-4-amino-4-deoxy-L-arabinose in Enterobacteriaceae (Grangeasse et al., 2003). *Wzc* has also been used as one of the marker genes for serotype identification in *K. pneumoniae*. The study by Hesse et al. (2020) showed that *wzc* mutation in CRKP prevented the infection of phages. Here, one of the selected phage-resistant strain hvKpP-R2 was identified as the *wzc* single-nucleotide mutation. We demonstrated the capsular differences between WT and mutant strains by TEM used in previous studies (Ernst et al., 2020; Li et al., 2021) and connected the phage resistance with bacteria virulence through the *wzc* gene in hvKp. Meanwhile, the results of other 17 *wzc* mutants showed that mutations at multiple sites of *wzc* gene were all capable of causing the resistance to phages and the decline of virulence. We believe that the significant reduction of virulence can have a certain effect on reducing the probability of liver abscess, soft tissue necrosis, etc., as well as reducing complications in patients with hvKp infection.

In addition, two independent groups found that *wcaJ* plays an important role in phage recognition and bacterial virulence, and all three groups had different mutation sites and patterns of *wcaJ* (Cai et al., 2019; Tan D. et al., 2020). In our study, we also found that the *wcaJ* mutation could restore phage sensitivity and virulence through complementary experiments (Supplementary Figure 4), indicating that the mutation frequency of *wcaJ* is high under phage selection pressure and demonstrating the important function of *wcaJ* in *K. pneumoniae* capsule synthesis. Here, a total of 33 of 45 phage-resistant isolates were determined to be *wzc* or *wcaJ* mutations, indicating that *wzc* and *wcaJ* were key factors in phage resistance of hvkp. *Wzc* and *wcaJ* are both involved in capsule synthesis, and so we audaciously predict that more genes involved in capsule synthesis will be identified in further studies of phage resistance and virulence changes.

## Conclusion

Taken together, the two new bacteriophages capable of lysing K57 capsular hypervirulent *K. pneumonia* were isolated. In the *in vivo* experiment, the bacteriophages were effective in treating bacterial infections in the *G. mellonella* larvae model. This study confirmed that the virulence of phage-resistant bacteria decreased as they developed resistance to phages. Further, it was verified that one or several base deletions of *wzc* and *wcaJ* genes played a role in phage receptor loss, resulting in no adsorption by the two phage strains and reducing virulence at the same time. This may explain why the phage-resistant bacteria did not affect the efficacy of treatment in *G. mellonella*. Those results suggest that hvKpP1 and hvKpP2 can be promising candidates for further investigation in phage-therapy research. As these phages targeted a hypervirulent serotype, and all their examined properties were suitable, our results may aid the development of bacteriophage-based therapeutic strategies for *K. pneumoniae* infections, specifically targeting hypervirulent strains.

## Data Availability Statement

The original contributions presented in the study are included in the article/Supplementary Material, further inquiries can be directed to the corresponding author/s.

## Author Contributions

LS, GL, and XJ contributed to the study design. XY, JH, and YX participated in strains collection. LS, XZ, GH, and YW carried out data analysis. LS and GL participated in the writing and revision of the article. XJ and GL provided project funds and were the authors of this article’s juxtaposition newsletter. All authors contributed to the article and approved the submitted version.

## Conflict of Interest

The authors declare that the research was conducted in the absence of any commercial or financial relationships that could be construed as a potential conflict of interest.

## Publisher’s Note

All claims expressed in this article are solely those of the authors and do not necessarily represent those of their affiliated organizations, or those of the publisher, the editors and the reviewers. Any product that may be evaluated in this article, or claim that may be made by its manufacturer, is not guaranteed or endorsed by the publisher.

## Footnotes

1
http://www.softberry.com

2
https://blast.ncbi.nlm.nih.gov
