# Supplementary material for: Phage Selective Pressure Reduces Virulence of Hypervirulent Klebsiella pneumoniae Through Mutation of the wzc Gene
Source: Front Microbiol. 2021 Oct 6;12:739319. doi: 10.3389/fmicb.2021.739319 (PMC8526901; doi:10.3389/fmicb.2021.739319)
Supplement: Supplementary file 1 [file Data_Sheet_1.DOCX]

Supplementary Material

# Supplementary Figures

**
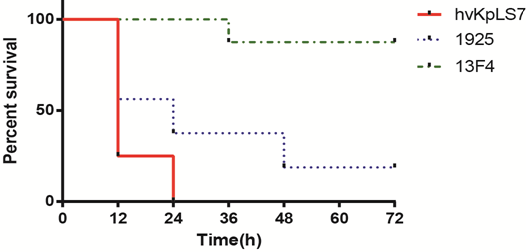
**

**Supplementary Figure 1.** Survival of *G. mellonella* after infection by the *K. pneumoniae* strains. The effect of 1 × 10^7^ CFU/ml of each strain on survival of *G. mellonella* is shown. hvKpLS7 was the host of phages, while 1925 and 13F4 were used as the control.


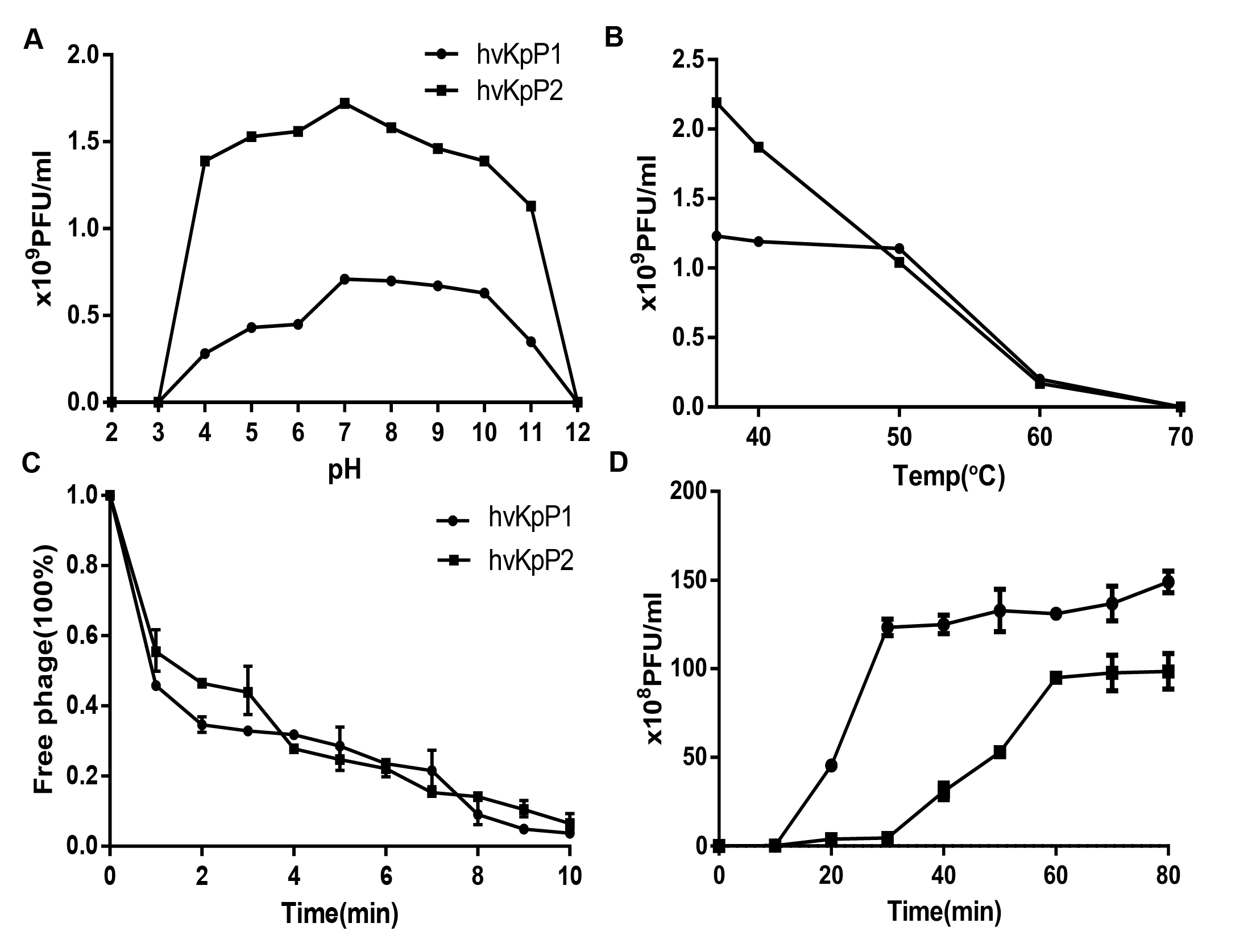


**Supplementary Figure 2.** (A) Survival rate of phages at different temperatures. (B) Phages stability at variable pH. (C) Adsorption rate. (D) One-step growth curve. *, P < 0.0001.





**Supplementary Figure 3.** **Phylogenetic analysis of whole genome.** The diagram was constructed using the MEGA7 program. The relative distances of each main branch are shown in the figure. Phage _Henu1, phage_117 and phage_31 can lyse hypervirulent *K. pneumonia*.


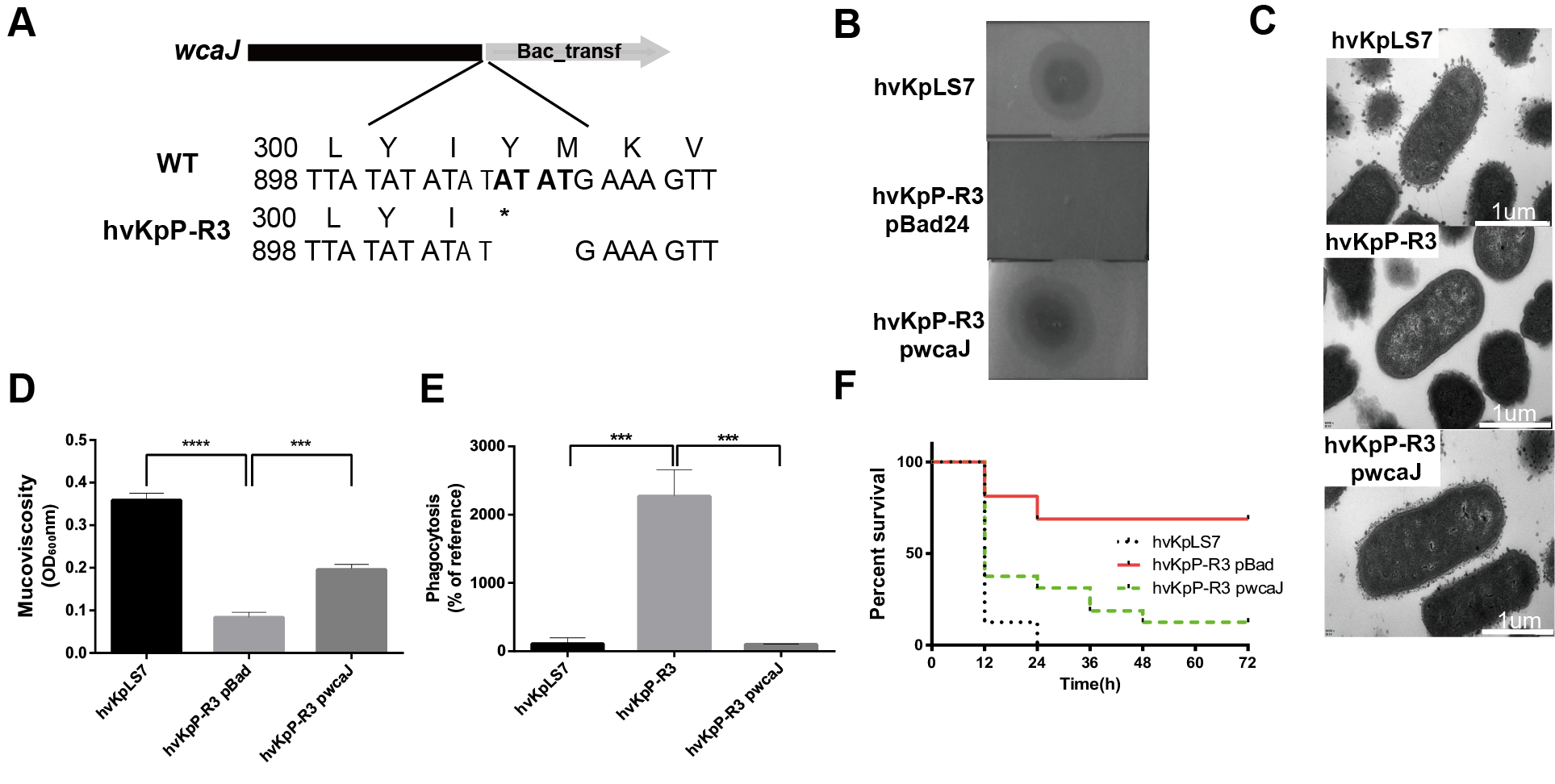


**Supplementary Figure 4.** The *wcaJ* mutant restore phage sensitivity and virulence by complementation. (A) Schematic representation of *wcaJ* in wild-type *K. pneumoniae* and hvKpP-R3. Genes are represented as arrows. (B) Spot test assay of phage on the parental *K. pneumoniae* strain hvKpLS7 and its derived mutants (hvKpP-R3 pBad and hvKpP-R3 pwcaJ). (C) The *wcaJ* gene affects capsule production. TEM of wild-type, phage-resistant and complementation strain. For every isolate, one representative image from six images obtained from one section is shown. (D) Mucoviscosity is restored in hvKpP-R3 pwcaJ. (E) Phagocytosis by RAW264.7 macrophages of wild-type, phage-resistant and *wcaJ* gene complementation strain. (F) Survival rates. Survival rates of *G. mellonella* with hvKpLS7, hvKpP-R3 pBad and hvKpP-R pwcaJ were determined. The one-way ANOVA test was performed to determine statistically significant differences. ***, P < 0.001; ***

# Supplementary Table

**Supplementary Table 1.** The sequences of primers

| **Genes** | **Primer** | | | **Sequence（5’ → 3'）** | |
| --- | --- | --- | --- | --- | --- |
| 27F | | F | AGA GTT TGA TCC TGG CTC AG | |  |
| 1492R | | R | AGA GTT TGA TCC TGG CTC AG | | |
| K1 | | F | GGTGCTCTTTACATCATTGC | | |
|  | | R | GCAATGGCCATTTGCGTTAG | | |
| K2 | | F | GACCCGATATTCATACTTGACAGAG | | |
|  | | R | CCTGAAGTAAAATCGTAAATAGATGGC | | |
| K57 | | F | CTCAGGGCTAGAAGTGTCAT | | |
|  | | R | CACTAACCCAGAAAGTCGAG | | |
| *ureA* | | F | GACAAGCTGTTGCTGTTTACC | | |
|  | | R | CGGGTTGTGAACGGTGAC | | |
| *wabG* | | F | ACCATCGGCCATTTGATAGA | | |
|  | | R | CGGACTGGCAGATCCATATC | | |
| *fimH* | | F | TGCTGCTGGGCTGGTCGATG | | |
|  | | R | GGGAGGGTGACGGTGACATC | | |
| *entB* | | F | ATTTCCTCAACTTCTGGGGC | | |
|  | | R | AGCATCGGTGGCGGTGGTCA | | |
| *ycf* | | F | ATCAGCAGTCGGGTCAGC | | |
|  | | R | CTTCTCCAGCATTCAGCG | | |
| *ybtS* | | F | CACCGCAAACGCAATCTG | | |
|  | | R | GCCATAGACGCTGTTGTTGA | | |
| *iutA* | | F | GGCTGGACATCATGGGAACTGG | | |
|  | | R | CGTCGGGAACGGGTAGAATCG | | |
| *aerobactin* | | F | GCATAGGCGGATACGAACAT | | |
|  | | R | CACAGGGCAATTGCTTACCT | | |
| *IroN* | | F | GGCTACTGATACTTGACTATTC | | |
|  | | R | CAGGATACAATAGCCCATAG | | |
| *KfuB* | | F | GAAGTGACGCTGTTTCTGGC | | |
|  | | R | TTTCGTGTGGCCAGTGACTC | | |
| *wcaG* | | F | GGTTGGKTCAGCAATCGTA | | |
|  | | R | ACTATTCCGCCAACTTTTGC | | |
| *alls* | | F | CCGAAACATTACGCACCTTT | | |
|  | | R | ATCACGAAGAGCCAGGTCAC | | |
| *uge* | | F | TCTTCACGCCTTCCTTCACT | | |
|  | | R | GATCATCCGGTCTCCCTGTA | | |
| *vatD* | | F | GAAGGAAACAAATCAGTA | | |
|  | | R | GTTTTATTTCGTTAGCAG | | |
| *rmpA* | | F | ACTGGGCTACCTCTGCTTCA | | |
|  | | R | CTTGCATGAGCCATCTTTCA | | |
| *gapA* | | F | TGAAGTATGACTCCACTCACGG | | |
|  | | R | CTTCAGAAGCGGCTTTGATGGCTT | | |
| *rpoB* | | F | GGCGAAATGGCGGAAAACCA | | |
|  | | R | GAGTCTTCGAAGTTGTAACC | | |
| *mdh* | | F | CCCAACTGCCTTCAGGTTCAG | | |
|  | | R | CCGTTTTTCCCCAGCAGCAG | | |
| *pgi* | | F | GAGAAAAACCTGCCGGTGCTGCTGGC | | |
|  | | R | CGCGCCAGGCTTTATAACGGTTAAT | | |
| *phoE* | | F | ACCTACCGCAACACCGACTTC | | |
|  | | R | TAATCAGAACTGGTAGGTCAT | | |
| *infB* | | F | CTCGCTGCTGGACTACATTCG | | |
|  | | R | CGCTTTCAGCTCAAGAACTTC | | |
| *tonB* | | F | CTTTATACCTCGGTACATCAGGTT | | |
|  | | R | ATTCGCCGGCTGAGCAGAGAG | | |
| pBad24-EcoRI-wzc-F | | F | TGGGCTAGCAGGAGGAATTCGTCGTCATCTATGGCTCGTC | | |
| pBad24-SalI-wzc-R | | R | TGCATGCCTGCAGGTCGACTACCAAGCCACACAACAGAC | | |
| pBad24-EcoRI-wcJj-F | | F | TGGGCTAGCAGGAGGAATTCATGACACTCTTTACAAAG | | |
| pBad24-SalI-wcaJ-R | | R | TGCATGCCTGCAGGTCGACTCAGTATGCACCATCTTTTT | | |

**Supplementary Table 2.** Summary of identified phage resistance mutations and tests of complementation.

| Mutation | Resistant isolates | Complementation |
| --- | --- | --- |
| *wzc* |  |  |
| 1273 A deletion(I425fs) | hvKpP-R7 | Yes |
| 1118 T deletion(L373ps) | hvKpP-R12, hvKpP-R13, hvKpPR-15, hvKpP-R25, hvKP-R27, hvKpP-R29, hvKpP-R30, hvKpP-R35, hvKpP-R42 | Yes |
| 253 C→T transversion(Q85ps) | hvKpP-R18, hvKpP-R37 | Yes |
| 1463 A deletion(N488fs) | hvKpP-R20 | Yes |
| 1133 IS1 family transposase insertion | hvKpP-R31, hvKpP-R32 | Yes |
| 807-895 deletion(E270fs) | hvKpP-R39 | Yes |
| 27-30 AACA deletion(Q9fs) | hvKpP-R40 | Yes |
| *wcaJ* |  |  |
| 910-911 AT deletion(M304fs) | hvKpP-R6, hvKpP-R8, hvKpP-R14, hvKpP-R17, hvKpP-R19, hvKpP-R21, hvKpP-R22, hvKpP-R28, hvKpP-R34, hvKpP-R36 | Yes |
| 903 T→A transversion(Y301ps) | hvKpP-R11 | Yes |
| 1268 C→T transversion(P423L) | hvKpP-R38, hvKpP-R41 | Yes |

fs, frameshift; ps, premature stop.
