# Supplementary figures and images for: Phage Selective Pressure Reduces Virulence of Hypervirulent Klebsiella pneumoniae Through Mutation of the wzc Gene
Source: Front Microbiol. 2021 Oct 6;12:739319. doi: 10.3389/fmicb.2021.739319 (PMC8526901; doi:10.3389/fmicb.2021.739319)

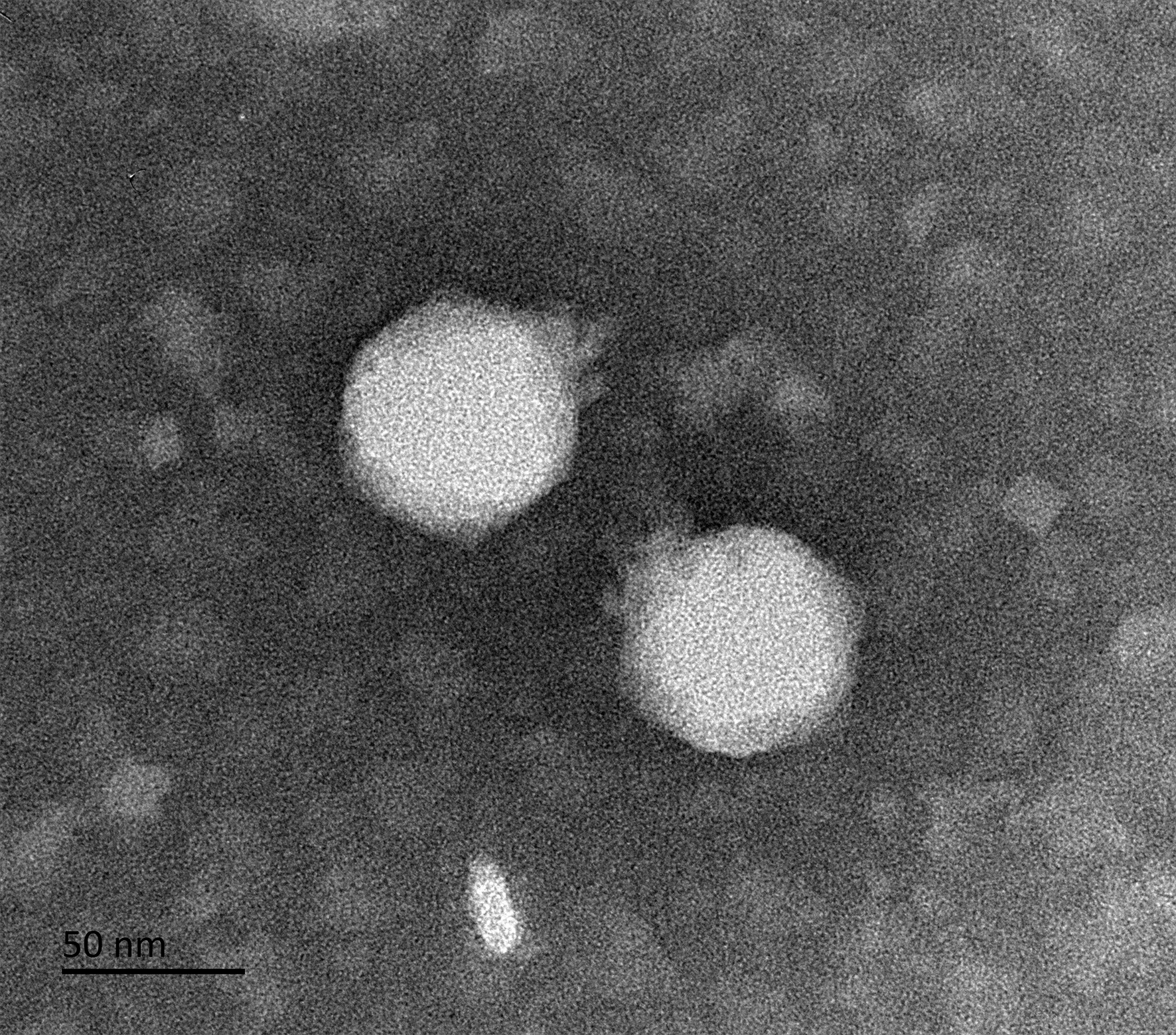

Supplement: Supplementary file 3 [file Image_1.TIF]

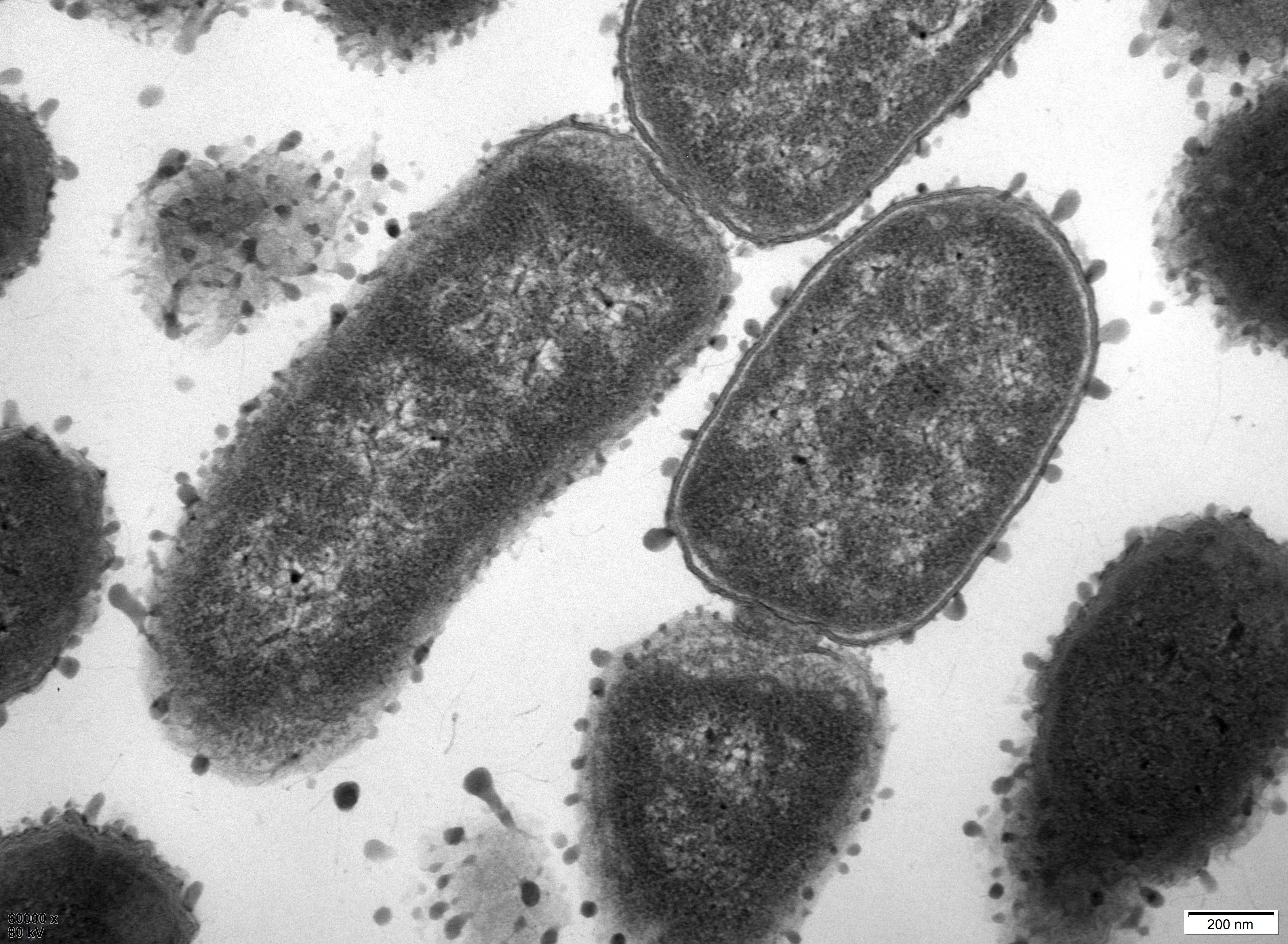

Supplement: Supplementary file 5 [file Image_3.TIF]

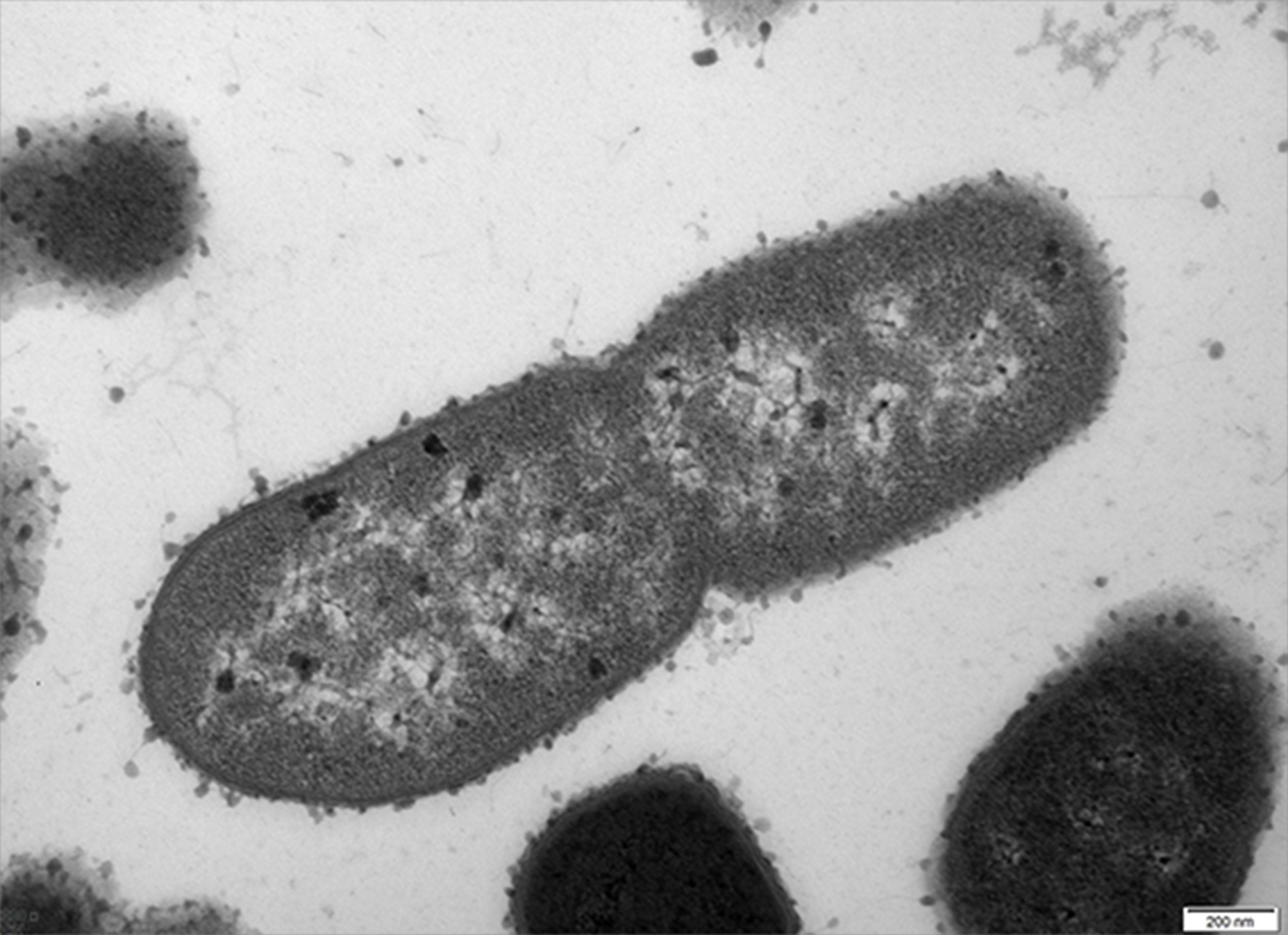

Supplement: Supplementary file 7 [file Image_5.TIF]

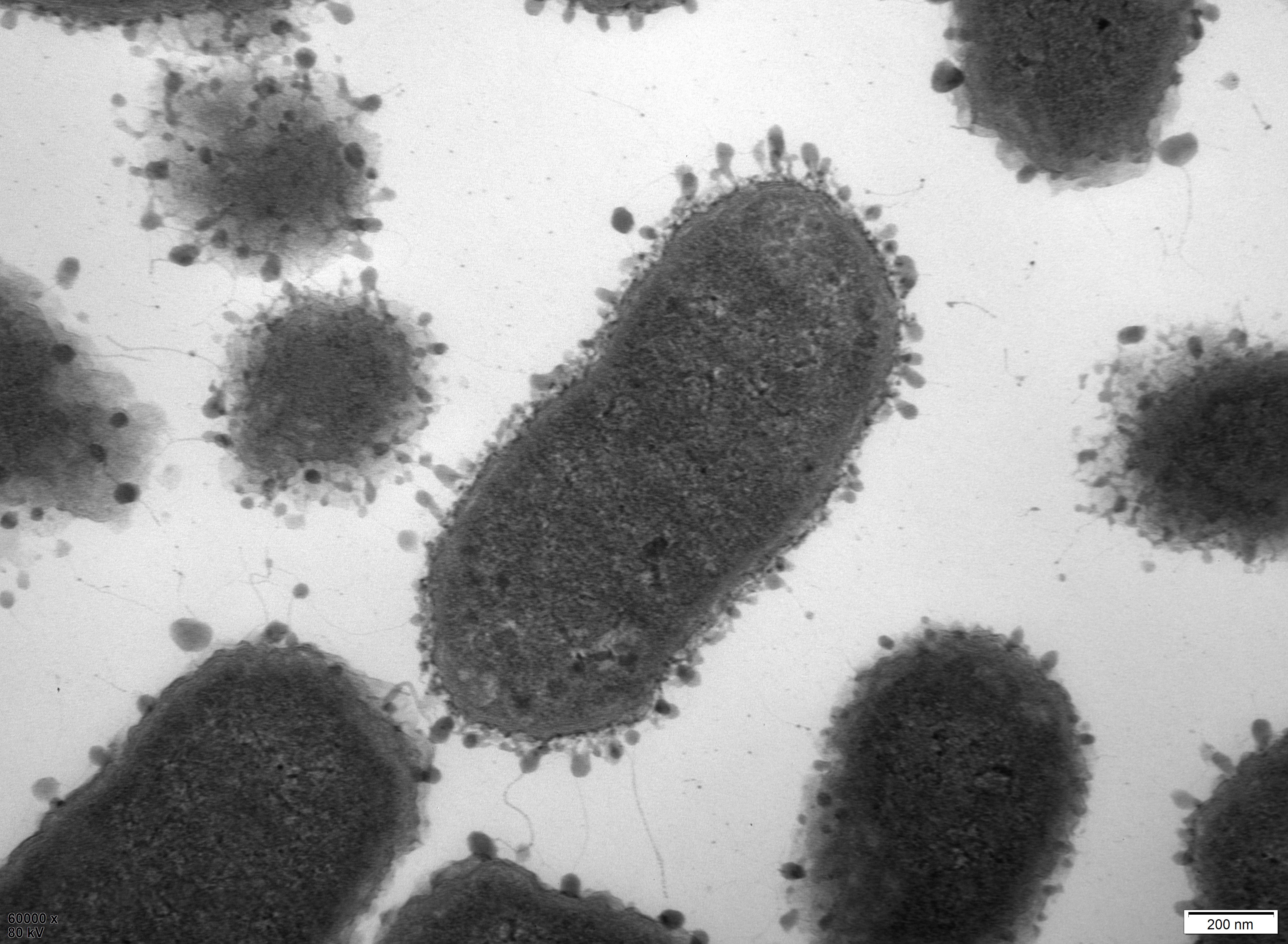

Supplement: Supplementary file 8 [file Image_6.TIF]
